# COPZ1: an example of non-oncogene addiction in human tumors

**DOI:** 10.3389/fphar.2025.1636326

**Published:** 2025-09-05

**Authors:** Tiziana Di Marco, Debora Vergaro, Angela Greco

**Affiliations:** Integrated Biology of Rare Tumors Unit, Experimental Oncology Department, Fondazione IRCCS Istituto Nazionale dei Tumori, Milan, Italy

**Keywords:** non-oncogene addiction, COPZ1, COPI, cancer target, cancer vulnerability

## Abstract

Non-oncogene addiction (NOA) indicates that tumor cell growth/survival requires the activity of genes/pathways not oncogenic per sé, and dispensable for normal cells. NOA genes provide a wide repertoire of novel therapeutic exploitable tumor vulnerabilities. A large body of evidence demonstrates the dependency of several tumors such as breast, prostate, ovary, thyroid, glioblastoma and LUAD, on the activity of COPZ1, a component of the heptameric COPI complex. Thus, COPZ1 is emerging as a potential novel therapeutic target for tumors of different origin. In different tumor models COPZ1 inhibition was found implicated in abortive autophagy, ER stress and activation of ferroptosis. In this review we summarize the different studies characterizing COPZ1 as a NOA gene in different tumor types, and discuss potential issues related to its targeting.

## 1 Introduction

**FIGURE 1:**
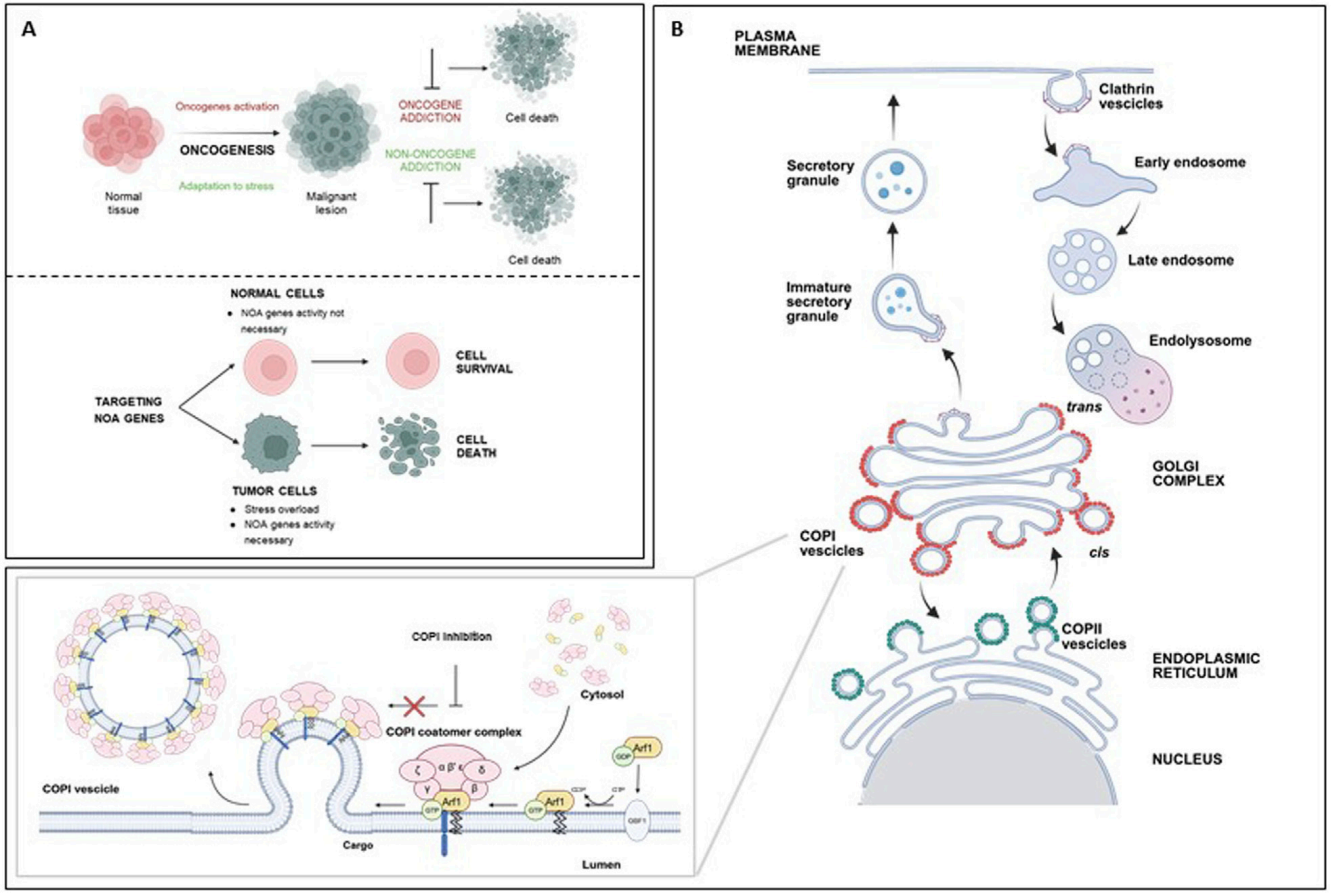
**(A)** top: Cancer cells rely not only on the activity of oncogenes (oncogene addiction), but also on the activity of normal genes, necessary to sustain the stress overload of the tumor phenotype (non-oncogene addiction); both represent targets for cancer therapy. **(A)** bottom: Targeting NOA genes impairs viability of tumor cells, while sparing normal cells. **(B)** Schematic representation of coated vesicles biogenesis in key intracellular trafficking pathways. Top: Clathrin-coated vesicles mediate endocytosis at the plasma membrane; they internalize extracellular material and membrane proteins into early endosomes, to be directed to degradation or recycling. Bottom: COPI-coated vesicles (red) mediate retrograde transport of proteins from the Golgi to the ER or intra-Golgi trafficking. The first step of COPI-vesicles formation (inset) is the conversion of Arf1-GDP in Arf1-GTP by GBF1. Arf1-GTP can then recruit the coatomer to the Golgi membrane. This complex, including seven subunits (greek letters), facilitates membrane curvature, cargo selection, and budding of the COPI-coated vesicles. This process is blocked by COPI targeting. COPII vesicles (green) mediate protein anterograde transport from ER to Golgi. ER, endoplasmic reticulum; COPI, coatomer complex I; COPII, coatomer complex II; Arf1, ADP-ribosylation factor 1; GBF1, Golgi Brefeldin A Resistant Guanine Nucleotide Exchange Factor 1; GDP, Guanosine Diphosphate; GTP, Guanosine Triphosphate. Created with Biorender.com.

As consequence of genetic alterations in oncogenes or tumor suppressor genes, cancer cells acquire a complex phenotype, including: uncontrolled proliferation and insensitivity to anti-growth signals, evasion of apoptosis, unlimited replicative potential, stimulation of invasion and metastasis, and induction of angiogenesis (Hanahan, 2022). The oncogene addiction concept (Figure 1A, top) indicates that tumor cells phenotype generally relies on the activity of specific driver alterations of oncogenes and related pathways (Weinstein, 2002), which to date have been considered the optimal targets for therapy (Weinstein and Joe, 2008). Indeed, the traditional approach for cancer target discovery is based on the identification of driver oncogenes through the characterization of the molecular alterations of primary tumors. A large variety of agents targeting oncogenes or their downstream pathways have been developed. Although successful at the clinic, target therapies present some limitations, such as modest and insufficient response, or the occurrence of resistance (Min and Lee, 2022; Zhong et al., 2021). This underlines the need of new strategies of intervention.

A complementary concept of cancer dependency is the non-oncogene addiction (NOA, Figure 1A, bottom) (Solimini et al., 2007). NOA concept indicates that, to sustain the stress caused by the uncontrolled growth, cancer cells are dependent on the activity of normal genes, that are not themselves oncogenes or otherwise mutated. Depletion of NOA genes impairs tumor cell viability, but has no effect on the growth of normal cells which are not similarly dependent on them (Figure 1A, bottom). NOA genes represent novel therapeutically exploitable cancer vulnerabilities. Of note, targeting NOA genes is expected to have limited side effects, since normal cells can tolerate their perturbation.

The canonical NOA concept (genes which are not classically or inherently oncogenic per sé as not mutated and expressed at physiological levels) has been subsequently broadened to include several intrinsic and extrinsic tumor dependencies necessary for tumor growth and surviving in the microenvironment. These include tumor cells overexpressed genes and intracellular signaling pathways, as well as genes/signaling axes functioning in non-cancer cells present in the microenvironment and fostering tumor growth (Hahn et al., 2021; Luo et al., 2009).

The traditional genetic approaches for cancer target discovery, based on the identification of gene lesions, transcriptional profiles, epigenetic modifications, are not suitable for the identification of NOA. The advent of loss-of-function RNA-interference-based genetic screenings, performed both *in vitro* and *in vivo*, has overcome this limitation, as they have the potential to unveil a genetic landscape for cancer vulnerabilities. Moreover, recently strategies, based on computational approaches, including statistical- and network-based as well as classic machine learning and deep learning methods, have broadened the number of NOA genes, and are useful when experimental models are not available (Sinha et al., 2017; Tsherniak et al., 2017).

Although the approaches aimed at uncovering NOA vulnerabilities are expected to provide a widened range of potential targets exceeding the number of known oncogenes (Hahn et al., 2021; Luo et al., 2009), most of them are not easily druggable or not considered enough attractive to enter drug development pipeline.

In this review we summarize the role of the coatomer complex I (COPI) in cancer, with specific interest to the COPZ1 subunit. We report different evidence supporting COPZ1 as a NOA gene in different tumor type; we discuss potential issues related to its targeting.

## 2 COPI complex

The human COPI coatomer (Figure 1B) is a hetero-heptameric protein complex involved in assembly of coated vesicles on Golgi membranes, retrograde transport of proteins in the ER-Golgi secretory pathway, endosome maturation, autophagy, viral infection, and lipid homeostasis (Bingham et al., 2024). The COPI complex comprises subunits such as coatomer subunit alpha (COPA), beta 1 (COPB1), beta 2 (COPB2), delta (ARKN1), epsilon (COPE), gamma 1 (COPG1), gamma 2 (COPG2), zeta 1 (COPZ1) and zeta 2 (COPZ2). The two gamma and zeta subunits are alternative in the COPI complex assembly. The COPI subunits are stably associated as a highly flexible complex in the cytosol and are recruited to Golgi membranes during vesicle biogenesis. The first step of this process is the activation of ADP-ribosylation factor 1 (Arf1) by the nucleotide exchange factor GBF1 on the Golgi membrane, converting it to its GTP-bound state. Arf1-GTP can then recruit the coatomer to the Golgi membrane. This complex facilitates membrane curvature, cargo selection, and budding of the COPI-coated vesicle. Following vesicle formation, GTP hydrolysis by Arf1 leads to coat disassembly, allowing fusion with the target compartment (Taylor et al., 2023).

Members of the COPI complex are involved in several human diseases, including cancer.

Loss of function mutations of COPI subunits are associated with different genetic diseases.

The inherited autoimmune disorder COPA syndrome (autoimmune interstitial lung, joint, and kidney disease) is caused by mutations of the coatomer complex member COPA, which impair ER-Golgi transport, increase ER stress, activate UPR, and trigger a chronic activation of the type I IFN response. The latter is due to a mislocalization and constitutive activation of STING to the Golgi apparatus (Deng et al., 2020; Lepelley et al., 2020; Taguchi et al., 2021; Watkin et al., 2015). Homozygous mutations in COPB1 cause Baralle–Macken syndrome, a rare genetic disorder characterized by global developmental delay, severe intellectual disability and cataracts (Macken et al., 2021). The R254C homozygous mutation in COPB2 causes primary autosomal recessive microcephaly 19 (MCPH19), a congenital brain malformation. In addition to MCPH19, COPB2 mutations also cause juvenile osteoporosis, and developmental delay (DiStasio et al., 2017; Marom et al., 2021). Mutations in COPD are linked to short stature–micrognathia syndrome, characterized by rhizomelic short stature and micrognathia (Ritter et al., 2022). A recent paper has identified COPZ1 gene as responsible for Severe Congenital Neutropenia Syndrome, characterized by defects in tissues of hematopoietic and neuronal origin. COPZ1 mutations, leading to loss-of function, were identified in two patients. In preclinical models the effects of COPZ1 mutations are rescued by ectopic expression of the paralogue COPZ2, which is expressed at low levels in hematopoietic and neuronal tissues (Borbaran Bravo et al., 2025).

The involvement of COPI in the replication of various viruses has been extensively documented. Notably, recent research documented the role of COPI complex in SARS-CoV-2 infection, specifically in the transport of virions from the ERGIC, thus suggesting COPI as a possible novel antiviral target (Hirabayashi et al., 2025).

COPI complex is involved in Alzheimer Disease (AD) pathogenesis. Bettayeb et al. (2016a) demonstrated *in vitro* that COPI subunit δ (δ-COP) regulates intracellular trafficking and maturation of amyloid precursor protein, and consequent production of Aβ peptides. The results were confirmed *in vivo* (Bettayeb et al., 2016b). In a mouse model of AD the reduction of δ-COP activity improved both the burden of plaques and plaque-related memory impairments. Furthermore, human genetic association studies identified genetic markers (SNPs) and mutations in COPI genes linked with an increased AD risk.

COPI complex plays a regulatory role on lipid droplet homeostasis. Depletion of COPI components resulted in lipid accumulation due to impaired lipolysis (Beller et al., 2008; Guo et al., 2008). According to a recent study, lipid accumulation is the consequence of ROS overproduction caused by COPI depletion (Gasparian et al., 2022). It is reasonable to speculate that COPI defects may account for metabolic diseases such as obesity and type 2 diabetes.

COPI members are involved in cancer. Recent reports have documented that different cancer type are dependent on the normal activity of COPI complex, proposing its targeting as a novel therapeutic approach. Targeting the coatomer complex, and consequently causing abortive autophagy and ER stress, may represent an optimal strategy to kill cancer cells, especially those unresponsive/resistant to conventional treatments.

Claerhout et al. have shown that targeting the COPI complex members in cancer cells of different origin is a potent approach to block productive autophagy and promote cell death (Claerhout et al., 2012). A recent study has shown that elevated expression of COPI subunits is commonly observed in intrahepatic cholangiocarcinoma (iCCA). In animal preclinical models of iCCA targeting COPI reduces tumor growth and triggers a CD8^+^ T cell mediated antitumor response, indicating a novel potential strategy for iCCA treatment (Chen et al., 2025). The co-occurrence of mutations in KRAS and LKB1 renders lung adenocarcinomas addicted to the ARCN1, COPB1, and COPA COPI complex members (Kim et al., 2013). By screening a siRNA library, we identified COPE and COPZ1 among a set of genes whose silencing inhibited the growth of a panel of thyroid tumor cells, but not of normal immortalized thyrocytes (Anania et al., 2015).

Several reports support the role of COPA in cancer. In mesothelioma, siRNA-based functional screening identified COPA as a potential therapeutic target, most likely related to its overexpression (Sudo et al., 2010). In cervical cancer COPA was proposed as a potential prognostic biomarker, based on the association of its moderate/strong expression with unfavorable prognosis. Moreover, COPA knock-down inhibits viability and tumorigenicity of cervical cancer cells, thus proposing it as a novel therapeutic target (Bao et al., 2022). In colorectal cancer (CRC) the A-to-I α-COP RNA editing by Adenosine deaminase acting on RNA 1 (ADAR1), which is highly expressed in some cancers, produces the COPA I164V mutation. The COPA I164V protein damaged the Golgi–ER reverse transport function, induced ER stress, UPR, and activates a transcription program leading to CRC cell invasion and metastasis (Wang et al., 2023). Urothelial cancer carrying FGFR aberration often develop resistance to the FGFR TK inhibitor Erdafitinib. A genome-wide CRISPR screen showed that targeting COPA enhances Erdafitinib sensitivity; functional validation allowed to propose COPA as a potential therapeutic target (Zhao et al., 2025).

COPB2 silencing was effective in reducing growth of human cholangiocarcinoma, gastric cancer, CRC, and lung adenocarcinoma cells as recently reviewed (Feng et al., 2021).

A functional screening identified COPB2 as a KRAS synthetic lethal partner in pancreatic cancer cell lines (Christodoulou et al., 2017). In breast cancer cell lines COPB2 upregulation was found to play a vital role, as its silencing inhibits proliferation and invasion, the latter through the decrease of EMT-related protein N-cadherin and vimentin (Mi et al., 2016). Similarly, knock-out experiments showed that COPB2 overexpression regulates PC-3 cell proliferation, cell cycle, and apoptosis (Bhandari et al., 2019). Saraon et al. demonstrated that targeting COPB2 through silencing or EMI66 represents a viable therapeutic option to attenuate mutant EGFR signalling and growth in non-small cell lung cancer (Saraon et al., 2021).

In asbestos-induced mesothelioma the somatic COPG1 C230R mutation was identified by exome sequencing and validated with a different approach (Mäki-Nevala et al., 2016).

A shRNA library screen identified ARCN1, the delta subunit of the COPI complex, as a new therapeutic target for cancer cells of different origins (Oliver et al., 2017).

High expression of COPZ2 gene is associated with poor prognosis and cancer progression in glioma (Geng et al., 2024).

The zeta 1 subunit, representing the topic of this review, will be treated in the next section.

## 3 COPZ1: a target for different tumor types

The COPI complex zeta 1 subunit is encoded by the COPZ1 gene on chromosome 12q13.13. COPZ1 participates to the COPI complex in alternative to COPZ2, encoded by the paralog gene on chromosome 17q21.32. A large body of evidence supports the dependency of several tumors on COPZ1 activity. Such “non-oncogene addiction” may involve either physiological- or over-expression of COPZ1 gene and represent an example of tumor proteostasis dependency.

Cancer cells dependency on physiological expression level of COPZ1 gene fulfils the classical NOA definition: “dependence of cancer cells on the normal functions of genes which are not classically or inherently oncogenic per sé as not mutated and expressed at physiological levels”. It was first described by Shtutman et al. (2011), who showed that tumor cells of different origin (i.e., prostate, breast, and ovarian carcinoma) are dependent on COPZ1, as its knockdown leads to cell death associated with Golgi apparatus collapse, ER stress, autophagy inhibition. Moreover, they showed that COPZ1 dependency is caused by the tumor-specific downregulation of the paralog gene encoding the COPZ*2* isoform, thus introducing the concept of paralog dependency. The evidence that COPZ1 knockdown kills both proliferating and non-dividing tumor cells, but spares normal cells, suggested the potential of COPZ1-targeting therapies to selectively eradicate cancer cells, independently of their proliferative status. Two important issues emerged from this study: 1) COPZ1 fits both canonical and paralog dependency definitions; 2) the downregulation of the paralog COPZ2 gene can predict the tumor response to COPZ1 inhibition.

Since 2015, we were interested in NOA in thyroid cancer (TC), with the final aim of identify novel vulnerabilities for TC cells. TC is the most common endocrine malignancy, generally associated with a good prognosis. The vast majority (90%) are differentiated tumors, papillary (PTC) and follicular (FTC) thyroid carcinomas, that can be effectively cured by standard therapies (Boucai et al., 2024). However, dedifferentiated thyroid cancers, such as anaplastic (ATC) and poorly differentiated (PDTC) carcinomas, although rare, have a dismal prognosis, as the available treatment options are unsuccessful. ATC is the rarest TC subtype, comprising only 2% of all cases; nevertheless, it accounts for up to 50% of TC- related deaths (Maniakas et al., 2022). The lack of effective therapies for aggressive TC highlight the need of identifying novel, unforeseen molecular targets. To face this need we performed the screening of a siRNA oligonucleotide library, and we identified a set of genes whose silencing inhibited the growth of a panel of human TC cells, while sparing that of normal immortalized thyrocytes. The COPZ1 gene was found among the top ranked genes, and was further validated.

We classified COPZ1 as an example of NOA for TC cells (Anania et al., 2015): its inhibition induced cell death in TC cell lines but not in immortalized normal thyrocytes; it was neither overexpressed nor mutated in TC. As for Shtutman study, thyroid tumors showed frequent downregulation of the paralog COPZ2 gene; these findings allowed us to speculate that the majority of TC may be eligible for COPZ1 targeting. We extensively characterized the mechanisms triggered by COPZ1 depletion in TC cells. We demonstrated that the reduction of viability upon COPZ1 depletion is associated with abortive autophagy, ER stress, unfolded protein response, and apoptosis. We demonstrated the efficacy of COPZ1 depletion in *in vivo* preclinical setting: in TC cells mouse xenografts local treatment with siRNAs targeting COPZ1 reduces tumor growth (Anania et al., 2017). More recently we demonstrated that COPZ1 depletion in TC cells may trigger an antitumor immune response. In fact, COPZ1 depletion activates a type I IFN response, which boosts a pro-inflammatory form of cell death able to promote dendritic cell maturation and subsequent activation of a T cell cytotoxic activity against parental tumor cells (Di Marco et al., 2020). Collectively, our findings support the notion that targeting COPZ1 may represent a promising therapeutic approach for TC, considering its specificity for cancer cells, the lack of effect on normal cells, and the capacity to prompt an anti-tumor immune response.

Several reports documented tumor dependency on COPZ1 overexpression. A pan-cancer bioinformatic analysis showed that high expression of COPZ1 was linked to poor overall survival in many cancers. Besides, the CRISPR Achilles’ knockout analysis revealed that COPZ1 was vital for many tumor cells’ survival (Hong et al., 2023).

Other studies have investigated the role of COPZ1 in different individual tumor types. In glioblastoma Zhang et al. have reported that COPZ1 overexpression is associated with tumor grade and poor prognosis. COPZ1 silencing suppressed the *in vitro* and *in vivo* growth of glioma cell lines; the involved mechanism includes upregulation of NCOA4, culminating in autophagy and ferroptosis. Interestingly, the authors unveil the involvement of COPZ1 in iron metabolism (Zhang et al., 2021).

A similar interplay between COPZ1 and NCOA4 and ferroptosis has also been reported in LUAD, a tumor type in which high expression of COPZ1 was indicative of malignancy and poor overall survival. Also, in LUAD COPZ1 depletion leads to *in vitro* and *in vivo* growth reduction of tumor cells (Wu et al., 2024).

COPZ1 is overexpressed in breast tumors with respect to normal tissue. COPZ1 knockdown inhibited proliferation, induced autophagy and promoted apoptosis of breast cancer cells. BMI1 was identified as a regulator of COPZ1 expression (Chen et al., 2022).

**FIGURE 2:**
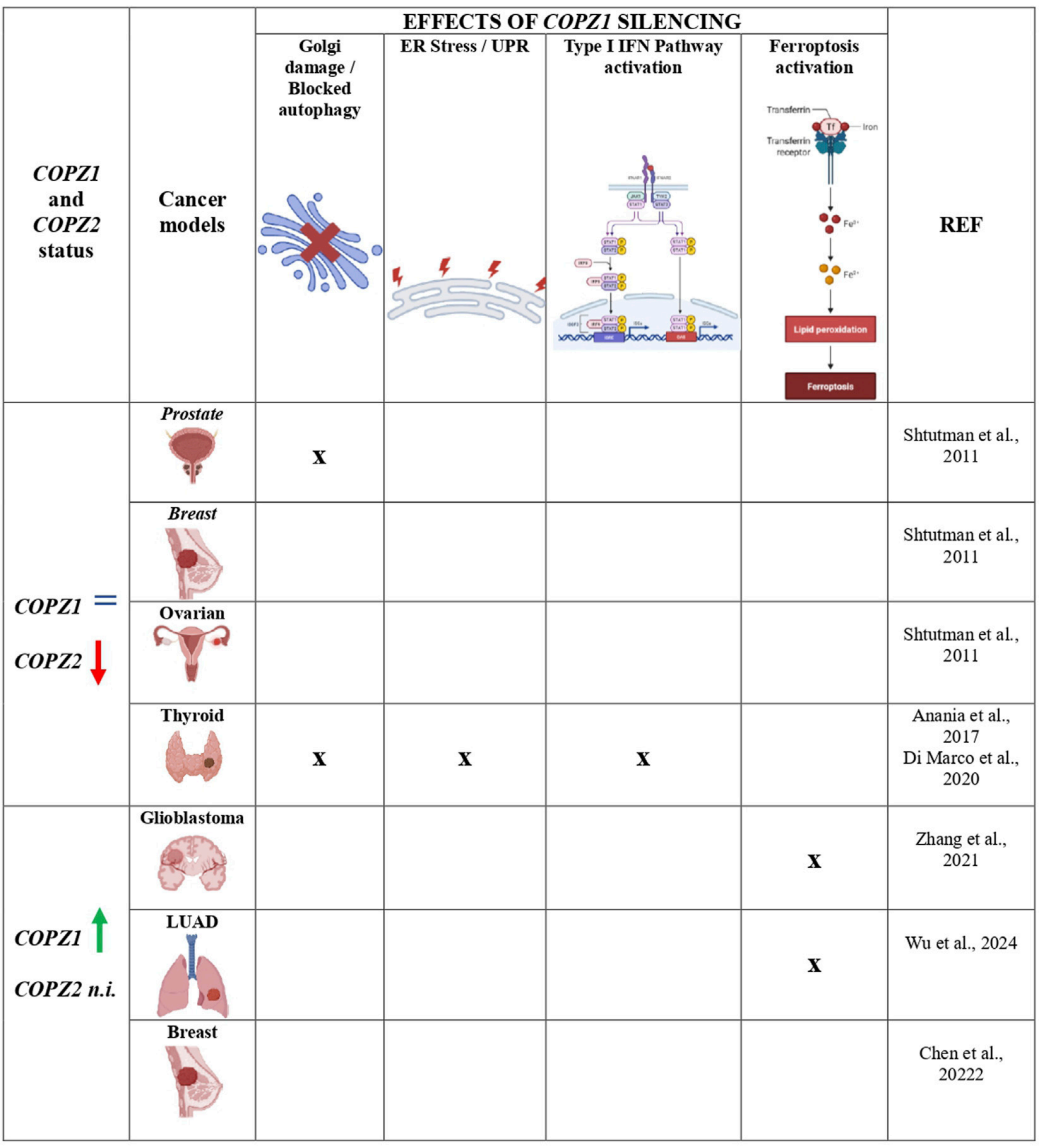
Mechanisms triggered by COPZ1 depletion in different tumor types. Summary of the principal intracellular mechanisms that are impaired and/or activated upon COPZ1 depletion. Regardless the status of COPZ1, the same of normal cells (=) or upregulated (green arrow), tumor cells die upon its depletion. In some tumor models it was found that the parolog gene COPZ2 is downregulated (red arrow): in this scenario tumor cell death is usually associated with Golgi apparatus impairment and blocked autophagy. In glioblastoma and LUAD, COPZ1 was found upregulated but the status of COPZ2 was not investigated (n.i.): in these two models cell death was found associated with activation of ferroptosis. The detection of the different mechanisms is indicated by X; empty cells indicate that they have not ben investigated. Created with Biorender.com.

Figure 2 summarizes the mechanisms triggered by COPZ1 depletion in different COPZ1-addicted tumor types.

At variance with Shtutman and our studies, in all the above studies COPZ1 was overexpressed. Moreover, none of the above studies assessed the status of COPZ2 whose expression, as mentioned above, is frequently downregulated in human cancer and can predict the vulnerability to COPZ1 depletion. Therefore, the role of COPZ2 in conferring COPZ1 dependency remains to be investigated.

Despite the numerous studies proposing COPZ1 as a possible target for different tumor types, COPZ1 inhibitors are not yet available. In principle, in order to interfere with ER functionality, they should abolish COPZ1 availability for the COPI complex formation. This can be achieved either interfering with its recruitment to the COPI complex, or blocking its interaction with other subunits, or inducing its degradation. Effort for the identification of small molecules targeting COPZ1 is ongoing. Recently, by meeting presentation Martins et al. proposed small molecules inhibiting COPZ1 in cancer cells (Martins et al., 2022; 2023). The Senex Biotech company is involved in the development of small molecules targeting COPZ1 (https://senexbio.com/other-cancer-targets/).

Sui et al. (2021) developed the Nano-ERASER technique for intracellular delivery and release of antibody, and degradation of a specific endogenous protein. They selected COPZ1 protein for validation, and showed that Nano-ERASER specifically degraded COPZ1 leading to cancer cell death without affecting normal cells.

Recently, by investigating the mechanism of action of the MCA-F3 antiviral compound, Li et al. demonstrated that it inhibits the binding of COPZ1 to EV-A71 non-structural protein 2C. The destruction of this interaction blocks the COPI coat-mediated transport of 2C to ER and ultimately inhibits EV-A71 replication (Li et al., 2024). The possible effect of COPZ1 inhibition by MCFA-F3 in cancer cells remains to be investigated. Considering that MCFA-F3 treatment neither reduce COPZ1 protein level, nor interferes with the correct COPI complex assembly, we consider unlikely its effect in COPZ1-addicted cancer cells.

Once identified, preclinical studies based on the inhibitor type will provide the rationale for their clinical translation.

An important issue to consider is the possible adverse effects of COPZ1 inhibition. According to the canonical definition of NOA (essential for cancer but not for normal cells) adverse effects of their inhibition are not expected. With respect to COPZ1, the susceptibility to its inhibition is related to the tumor-specific downregulation of the paralog gene COPZ2. As consequence, no adverse effects are expected. This issue needs to be revised, based on recent data. In patients with severe congenital neutropenia syndrome, point mutations in COPZ1 gene have been identified, and functionally classified as loss of function (Borbaran Bravo et al., 2025). The clinical manifestations involve tissues of hematopoietic and neuronal origin, which express very low levels of COPZ2, at variance with the remaining human tissues. The authors showed in preclinical models that the defective granulopoiesis caused by COPZ1 mutations can be rescued by ectopic expression of COPZ2 or treatment with a HIF1α stabilizer. Translating this new evidence in COPZ1-addicted tumors, adverse effects of COPZ1 targeting in hematological and neurological tissues should be expected and considered. Future investigations should explore strategies for increasing COPZ2 levels in hematological and neurological tissues, in order to mitigate COPZ1-depletion side effects.

Another issue deserving further investigation is the possible integration of COPZ1 targeting with standard cancer treatment; it would be important to assess if COPZ1 targeting might enhance the sensitivity of cancer cells to conventional therapeutic agents.

An interesting implication is the capacity of COPZ1 inhibition to trigger a *in vivo* T cell mediated antitumor response. This was documented by us in TC preclinical models (Di Marco et al., 2020), but in other COPZ1-addicted tumor types remains to be investigated. On the other hand, the activation of type I IFN signalling is common feature for COPI inhibition. It has been reported that COPB1-knockdown induced type I IFN signaling activation, leading to inhibition of *Chlamydia psittaci* intracellular proliferation (Li et al., 2025); pathogenic COPA variants cause chronic activation of the type I IFN signaling (Deng et al., 2020; Lepelley et al., 2020; Taguchi et al., 2021; Watkin et al., 2015); in iCCA targeting COPⅠ activates STING-IFN-Ⅰ pathway, triggering an anti-tumor T cell response. This evidence allows speculating that immunogenic response might represent a general effect of COPZ1 inhibition in tumors of different type. This issue deserves to be addressed and has important therapeutic implications, as it would strengthen the validity of COPZ1 as antitumor target. In addition to a direct effect on tumor growth, COPZ1 targeting may have potent immune-stimulatory effects through the establishment of a systemic inflammatory response, thus turning “cold” immunosuppressive tumors into “hot” inflamed tumors. The restoration of the immune regulatory properties against tumor cells might improve the efficacy of immunotherapy, as well as chemotherapy and radiotherapy.

## 4 Conclusion

Mounting evidence support COPZ1 as a NOA gene in different tumor types, proposing it as a potential novel therapeutic targets for tumors of different origin. However, no specific COPZ1 inhibitors have been yet identified. We believe that the increasing number of tumor types addicted to COPZ1, together with the possibility that COPZ1 targeting may evoke an antitumor immune response, further support the importance and need of identifying specific COPZ1 inhibitors. In order to corroborate the validity of COPZ1 as tumor target, further efforts should also be dedicated to assess if the effects of COPZ1 inhibition are context-specific or shared by different tumor type models, as well as to validate COPZ2 as a biomarker for patient stratification.
